# The Digital Maze Test Offers a Unique Method for Detecting Early Cognitive Changes in Preclinical Alzheimer’s Disease

**DOI:** 10.1002/alz.095237

**Published:** 2025-01-09

**Authors:** Grace A Del Carmen Montenegro, Talia L Robinson, Jessie Fanglu Fu, Michael J Properzi, Dana Penney, Randall Davis, Reisa A Sperling, Keith A Johnson, Rebecca E Amariglio, Dorene M Rentz

**Affiliations:** ^1^ Massachusetts General Hospital, Boston, MA USA; ^2^ Brigham and Womens Hospital, Bostion, MA USA; ^3^ Department of Neurology, Massachusetts General Hospital, Harvard Medical School, Boston, MA USA; ^4^ Massachusetts General Hospital, Harvard Medical School, Boston, MA USA; ^5^ Lahey Hospital and Medical Center, Lexington, MA USA; ^6^ MIT Computer Science And Artificial Intelligence Laboratory, Cambridge, MA USA

## Abstract

**Background:**

Digital cognitive tools offer novel ways to detect early cognitive changes associated with preclinical Alzheimer’s disease (AD). A digital version of the maze test (dMaze) was recently developed using a digital pen (12 ms temporal precision) to uniquely capture the process of test completion, reflecting the thinking effort, potentially more sensitive to early cognitive deficits in preclinical AD. The sensitivity of these novel digital maze test variables—Wall Penetration Count and Speed Standard Deviation (Speed SD)—to detect early amyloid‐β burden was evaluated, hypothesizing that greater amyloid accumulation would be associated with greater variability of speed, and more wall penetration errors while completing the task, reflecting a greater thinking effort.

**Method:**

A cohort of 221 participants (CN = 208, MCI = 9, Dementia = 4) completed dMaze pairs possessing the same motor solutions but differing in cognitive loads: simple path following “no‐choice” (NC) and “choice” (CH) containing decision points that necessitate selecting path alternatives, thus adding elements of strategic problem‐solving. Wall Penetration Count tallied invasions into maze boundaries, and Speed SD quantified speed variability. Global amyloid‐β burden was assessed using [^11^C]Pittsburgh‐Compound‐B (PiB) PET, estimated as distribution volume ratio (DVR) in neocortical aggregate regions. Separate linear regression models examined the associations between the digital maze test variables and global amyloid‐β, adjusting for age and education. Analyses were conducted for NC and CH conditions separately.

**Result:**

In the CH conditions, higher amyloid‐β burden was significantly associated with a higher Wall Penetration Count (b = 2.26, p = 0.03, R^2^ = 0.03) and greater Speed SD (b = 1.65, p = 0.04, R^2^ = 0.20). In the NC conditions, Wall Penetration Count and Speed SD were not significantly associated with amyloid‐β burden(b = 1.10, p = .12; (b = 0.99, p = .23).

**Conclusion:**

Speed variability and wall penetration errors in the completion of the more challenging maze test choice condition were associated with higher amyloid‐β burden in a largely cognitively normal sample. This association was not observed in the NC conditions, suggesting that the higher cognitive demands of the CH mazes may unmask subtle, amyloid‐related disruptions in navigational strategies. These findings support the potential utility of the dMaze tests as sensitive tools for detecting early pathophysiological deficits in AD.

